# A Functional Adenosine Deaminase Polymorphism Associates with Evening Melatonin Levels and Sleep Quality

**DOI:** 10.5334/jcr.209

**Published:** 2021-04-28

**Authors:** Jaime L. Tartar, Franklin S. Hiffernan, Kristine E. Freitas, Ana I. Fins, Jonathan B. Banks

**Affiliations:** 1 Department of Psychology and Neuroscience, Nova Southeastern University, Ft. Lauderdale, FL, USA; 2 Department of Clinical Psychology, Nova Southeastern University, Ft. Lauderdale, FL, USA

**Keywords:** adenosine, adenosine deaminase, melatonin, mood, sleep

## Abstract

Increased adenosine levels throughout the day promote sleepiness. A single nucleotide polymorphism (SNP) in the adenosine deaminase ADA gene (rs73598374) has been shown to affect sleep regulation. The extent to which lower ADA enzymatic activity is associated with the homeostatic sleep factor, melatonin, is uncertain. To test this possibility, we assessed the relationship between the ADA polymorphism and evening melatonin levels, as well as self-reported sleep behavior. Given the close relationship between mood and sleep behavior, we further tested the impact of ADA genotype on self-reported mood. We show that relative to the GG homozygotes, the A allele carriers (higher adenosine levels) had significantly higher evening melatonin levels as well as significantly better sleep quality. We further show the correlations between sleep and mood measures were altered by ADA genotype, with a stronger relationship observed in the GG (lower adenosine) group. Combined, these findings advance our understanding of the biochemistry of melatonin production by showing that there is a relationship between ADA genotype and melatonin levels. The differential relationships between sleep and psychological health between the genotype groups may reveal novel insights about the development of genotype-specific progression of various psychological disorders such as chronic anxiety and stress.

## Introduction

The purine nucleoside adenosine has been clearly established as a somnogenic product of adenosine triphosphate (ATP) degradation: extracellular adenosine levels in the basal forebrain increase during the day or with prolonged wakefulness and decline with subsequent sleep [1,2,3]. Adenosine deaminase (ADA) is responsible for metabolizing adenosine to inosine and functional changes in the ADA gene can affect ADA enzyme activity. In particular, a functional G to A single nucleotide polymorphism (SNP) at nucleotide 22 of exon 1 in the ADA gene changes Asp to Asn, which modulates the activity of the ADA enzyme (rs73598374). Carriers of the ADA A allele have lower enzymatic activity, and consequently, higher levels of circulating and intracellular adenosine levels relative to individuals who are homozygous for the G allele [4,5,6]. Converging lines of evidence demonstrate a role for this ADA polymorphism in sleep behavior wherein carriers of the AG polymorphism (decreased ADA enzymatic activity) show increased amounts of slow wave sleep and a reduced number of nocturnal awakenings [7,8,9]. However, the mechanisms through which ADA activity can influence sleep are currently uncertain.

One largely unexplored mechanism through which ADA can affect sleepiness is through its ability to alter melatonin synthesis in the pineal gland. The absence of light (signaled through the retinohypothalamic tract) increases melatonin release which increases sleepiness [10]. Melatonin synthesis occurs through the conversion pathway from tryptophan to 5-hydroxytryptophan (5-HTP) to 5-hydroxytryptamine (5-HT), to N-Acetylserotonin (NAS), and finally, into melatonin. Direct administration of adenosine onto rat pineal extracts has been shown to result in a 3–4 fold increase in melatonin release [11]. Further underscoring the relationship between adenosine and melatonin, ATP has been shown to increase NAS synthesis in rat pineal glands though an inhibition of acetylserotonin O-methyltransferase (ASMT) [12]. Since adenosine is a metabolite of ATP, extended wakefulness will expend large amounts of ATP, resulting in adenosine accumulation. It is possible, therefore, that differential expression of ADA will affect not only circulating adenosine levels, but also melatonin production. Indeed, a previous study with only 24 participants (12 GA, 12 GG allele carriers) demonstrated that not only did the GA allele carriers feel sleepier than the GG carriers, but they also had higher melatonin levels under low sleep pressure conditions [13]. To further test the uncertainty surrounding melatonin levels and the ADA polymorphism, the current study sought to test the central hypothesis that ADA A allele carriers (higher adenosine levels) would have higher evening melatonin levels. Given the critical role that adenosine and melatonin play in sleep and wakefulness, and the demonstrated role of ADA genotype in sleep depth, we also examined self-reported measures of sleep health. We predicted that, in agreement with the previously reported EEG findings and our central hypothesis, that ADA A allele carriers would have better self-reported sleep health across all measures.

We also tested a secondary hypothesis that the ADA genotype would relate to psychological health measures. Given the close link between sleep loss and mood disorders, it is critical to examine psychological health when measuring sleep behavior, however, we also thought it possible that the ADA genotype would directly relate to psychological health. Previous research has shown that adenosine plays a role in not only sleep regulation, but also mood and social interaction. This idea was first proposed almost a century ago with the finding that mood impairments were associated with a decrease in uric acid secretion [14]. Since then, it has been discovered that, relative to a control group, there is decreased serum ADA activity in major depressives [15] and A2A receptor antagonism and deletion ameliorate the symptoms of behavioral despair in mice [16]. In addition, A2A receptor antagonism reverses behavioral and physiological stress responses after maternal separation in rats [17]. Indeed, purinergic intervention has even been suggested as a potential therapeutic intervention for mood disorders [18]. We predicted that, in agreement with previous work, relative to the AG genotype group, the GG genotype group would report better psychological health.

## Materials and Methods

### Participants

One hundred and fourteen participants (mean age of 23, SD = 6.89, 82 women) were recruited through flyers posted in public buildings and through the Nova Southeastern University (NSU) introductory psychology course participant pool. Exclusion criteria during study enrollment included being younger than 18 years of age or over 50 years of age, having a positive history of mental illness, taking medication for sleep, or a diagnosed sleep disorder. All participants were compensated with either a $10 store gift card or research participation credit. Participant testing was carried out in accordance with the Declaration of Helsinki and a study protocol approved by the Institutional Review Board at Nova Southeastern University (2015142-NSU). All participants received a verbal explanation of the study procedures and signed an NSU IRB-approved written Informed Consent Form.

### Procedure

All participants signed a written consent form, provided two saliva samples (one for DNA extraction and one for melatonin quantification), and completed a series of psychological instruments to measure sleep and mood. We assessed sleep quality through the Pittsburgh Sleep Quality Index (PSQI), insomnia through the Insomnia Severity Index (ISI), and sleepiness through the Epworth Sleepiness Scale (ESS). We also used the Morningness-Eveningness Questionnaire (MEQ) to ensure that there was not a chronotype difference between the genotype groups. We further tested the extent to which ADA genotype was related to a range of psychological health measures including, perceived stress (perceived stress scale, PSS), state and trait anxiety (state trait anxiety inventory, STAI), depression symptomology (CES-D), and mood disturbance (profile of mood states, POMS).

Testing occurred between 6:00–8:00 p.m.- a time when melatonin is starting to rise. Saliva was collected at the end of testing after participants sat in a dimly lit room (60 lux) for 30 minutes. Participants provided saliva samples for melatonin quantification and DNA extraction via passive drool though a straw into 1.5 mL micro-centrifuge tubes while they completed the inventories.

#### Sleep Assessments

##### Pittsburgh Sleep Quality Index (PSQI)

Sleep quality was measured using the self-rated PSQI questionnaire [19]. The PSQI is a 19-item instrument that yields seven component scores in addition to a global score of sleep quality. With strong psychometric properties, the instrument is used widely. The instrument exhibits high internal consistency (Cronbach α = 0.83), adequate test-retest reliability scores across the sleep-related components of the instrument for a non-clinical sample (from 0.44 to 0.70) and the ability to differentiate controls from sleep-disordered groups [19]. Internal consistency for the sleep-related components was adequate in the current sample (Cronbach α = .68).

##### Epworth Sleepiness Scale (ESS)

The ESS was utilized to evaluate the degree of daytime sleepiness. The scale consists of 8 items that evaluate the propensity to fall asleep in a variety of daytime activities [20]. In a sample of college students 18–25 years old, internal consistency was adequate (α = 0.71) [21]. Test-retest reliability has been estimated to be 0.82 [22] and, supporting its construct validity, the measure is sensitive to changes in sleepiness among individuals with narcolepsy being treated with modafinil [20]. Internal consistency was adequate in the current sample (Cronbach α = .71).

##### Insomnia Severity Index (ISI)

The ISI was employed to assess insomnia symptoms and their impact on daily functioning [23]. The seven-item instrument measures several dimensions associated with insomnia, such as sleep onset, sleep maintenance and early morning awakening difficulties as well as daytime dysfunction. Its reliability with a community (non-clinical) sample is high with Cronbach α = 0.90 [24]. Significant correlations with other measures of fatigue, quality of life and mood support its convergent validity; criterion validity is supported by the ability of the instrument to correctly classify a large sample of community-dwelling individuals [24]. Internal consistency was acceptable in the current sample (Cronbach α = 84).

#### Psychological Health Inventories

##### State-Trait Anxiety Inventory (STAI-Y)

State and trait anxiety were measured using the STAI-Y [25]. The Trait and State scales each consist of 20 items. The instrument has been used extensively in research and clinical practice. Spielberg et al. [25] report internal consistency coefficients for young adult females to be 0.93 for State anxiety and 0.92 for Trait anxiety. Test-retest reliability coefficients range between 0.65 to 0.75 [25]. Moreover, it has been validated as an accurate measure of anxiety in adults [26] and convergent and discriminant validation has been exhibited when compared with other measures [27]. Internal consistency was acceptable in the current sample for both state (Cronbach α = .86) and trait scales (Cronbach α = .84).

##### Profile of Mood States (POMS)

The POMS was applied in the current study to measure acute and ongoing mood [28]. It consists of 65 items that tap six scales assessing anger-hostility, confusion-bewilderment, depression-dejection, fatigue-inertia, tension-anxiety and vigor-activity in addition to a composite score of total mood disturbance. Internal consistencies vary from 0.84 for the confusion-bewilderment scale to 0.95 for the depression-dejection scale while test-retest reliabilities range from 0.65 for vigor to 0.74 for depression [29]. McNair et al. [29] also provide supportive evidence of the instrument’s criterion-related validity through a variety of studies. Internal consistency was acceptable in the current sample (Cronbach α = 88).

##### The Center for Epidemiologic Studies Depression Scale (CES-D)

The CES-D was employed to measure depressive symptomatology [30]. Unlike other depression scales that focus on clinical populations, the CES-D was created to be utilized with the general population. Twenty items are rated on a 4-point Likert scale. In a college sample, Cronbach α was found to be 0.87 [31]. Radloff [30] reported moderate test-retest correlations ranging from 0.32 (one-year retest interval) to 0.68 (four-month interval). The instrument is also able to accurately discriminate between patient and non-patient groups [31]. Internal consistency was adequate in the current sample (Cronbach α = .85).

##### Morningness-Eveningness Questionnaire (MEQ)

We determined a possible influence of a gentoype influence of chronotype using MEQ scores [32]. This instrument contains 19 items which tap an individual’s waking and bed times, preferred times for engaging in a variety of activities and subjective alertness at sleep and wake times. Internal consistency is good, with a Cronbach α = 0.82 [33]. Test-retest reliability correlations across a number of studies have been reported to range between 0.84 and 0.95 and the instrument has been validated against a variety of circadian-related variables such as body temperature, melatonin and cortisol secretion and sleep habits [34]. Internal consistency was acceptable in the current sample (Cronbach α = .70).

##### Perceived Stress Scale (PSS)

The 10-item PSS was applied to measure current stress levels in the participants and as a complement to cortisol measures It exhibits acceptable internal consistency with Cronbach α’s ranging from 0.78 to 0.91 [35]. Construct validity has been demonstrated via the relationships between the instrument and various measures of stress and sources of stress as well as measures of health and health behaviors [35]. Internal consistency was adequate in the current sample (Cronbach α = .83).

#### Biomarkers

##### Melatonin

Saliva samples were run in duplicate and quantified via a human melatonin enzyme immunoassay (EIA) kit per the manufacturer’s instructions (Salimetrics LLC, USA). The samples were immediately read in a BioTek ELx800 plate reader (BioTek Instruments, Inc., USA) at 450 nm with a correction at 630 nm. All samples were within the detection ranges indicated in the melatonin immunoassay kit and the variation of sample readings was within the expected limits. Final concentrations for the biomarkers were generated by interpolation from the standard curve in pg/mL.

##### Genotyping

Genomic DNA was extracted in a QIAcube instrument following the manufacturer’s standard protocol for saliva nucleic acid extraction (QIAGEN, Valencia, CA). After isolation, allelic discrimination for the ADA gene was determined via real-time polymerase chain reaction (PCR) using a TaqMan SNP genotyping assay using fluorogenic probes (Applied Biosystems, CA). Thermal cycling was performed on StepOne Real-Time PCR system (Applied Biosystems, CA). The amplification mix contained the following ingredients: 12.5 μL of PCR master mix (QIAGEN, Valencia, CA), 1.25 μL of TaqMan 20X working stock, 10.25 μL of RNase- and DNase-free water (Sigma), and 1.0 μL of sample DNA, in a total volume of 25 μL per single tube reaction. The PCR conditions were 95 °C for 10 minutes followed by 40 repeated cycles of 95 °C for 15 seconds and 60 °C for 60 seconds. Genotypes were determined automatically via the StepOne software (Applied Biosystems, CA) based on the fluorescence signals. Samples were run in duplicate and in the case of a call discrepancy, samples were rerun.

### Statistical Analyses

The distributions of allele frequencies were determined by the Hardy–Weinberg exact test, and the association of allele were analyzed using the chi-square test. All calculations were conducted using the R system for statistical analyses [36]. All reported p-values are two-tailed with a priori significance level of p < 0.05. To test the differences between the ADA genotypes a series of t-tests was conducted. To further test the hypothesis that ADA genotype may alter psychological health, we examined the correlations between melatonin and the psychological health variables in each genotype separately. To test for differences between the strength of the correlations in each group Fisher’s z tests were conducted using the ConCor [37] package and 95% confidence intervals around the difference between the correlations are reported [38].

## Results

**Figure 1:**
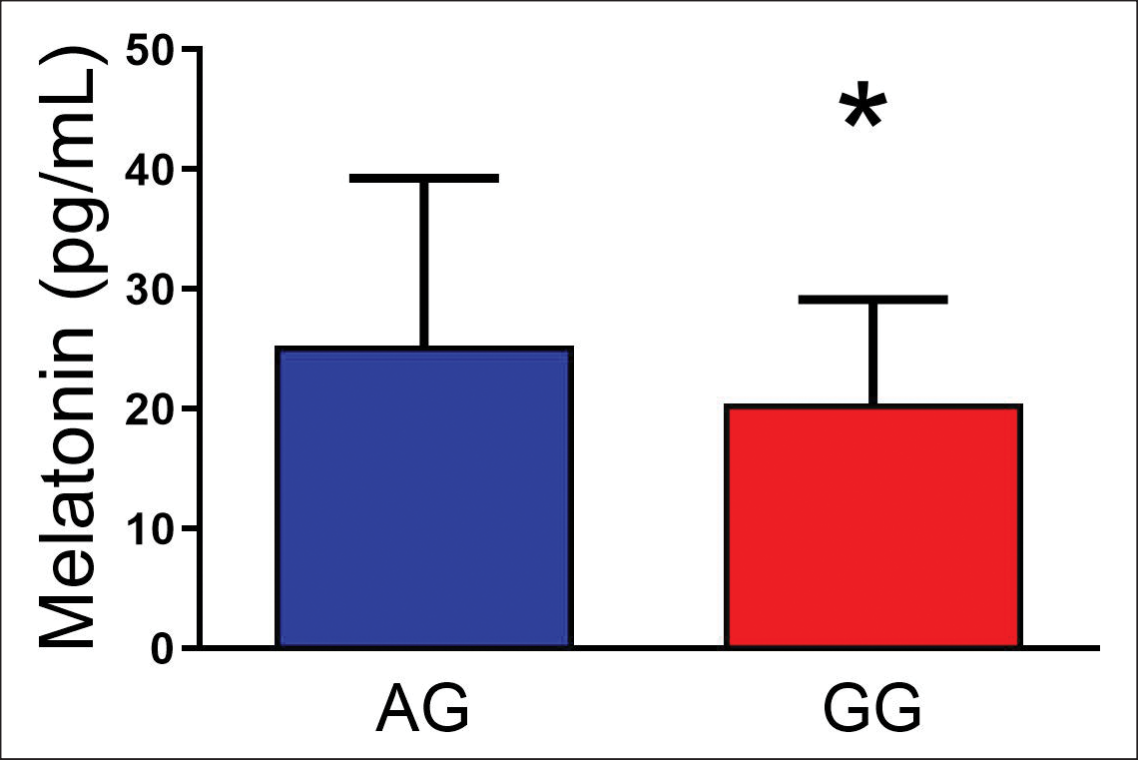
Evening melatonin levels according to genotype. There was a significant effect of ADA genotype on evening melatonin levels. Higher melatonin levels were measured in the AG genotype group (less enzymatic activity, higher adenosine) relative to the GG genotype group. Data are means ± SD, * = p < 0.05.

To test our central hypothesis, that ADA genotype would impact melatonin levels, we conducted a t-test. As seen in ***Figure 1***, significant differences were observed between the AG and GG genotypes on melatonin, *t* (117) = –2.51, *p* =.024, *d* = 0.486, with lower melatonin levels in the GG group. This difference is not likely due to chronotype differences since the two groups were similar on the MEQ measure (see ***Table 1***). To test the hypothesis that the ADA genotype would alter sleep quality we conducted a series of t-tests examining differences between ADA genotype on the PSQI, ISI, and ESS. A significant difference between AG and GG allele carriers was observed on the PSQI Global score, *t* (115) = 2.38, *p* = .019, *d* = 0.465, with the AG allele carriers reporting lower scores, as seen in ***Table 1***. However, no significant differences were observed on the ISI or ESS.

**Table 1:** Descriptive Information for all variables by ADA group.


	AG GENOTYPE MEAN (*SD*)		GG GENOTYPE MEAN (*SD*)		t	p

Melatonin (pg/mL)	25.27	13.98	20.45	8.68	-2.51	**.014**

Perceived Stress (PSS)	17.00	9.02	14.29	6.12	-1.91	.058

State Anxiety (STAI)	32.08	12.22	31.91	8.54	-0.08	.931

Trait Anxiety (STAI)	39.13	9.12	36.05	8.35	-1.81	.072

Depressive symptomatology (CESD)	8.78	6.92	9.07	6.79	0.21	.837

Profile of Mood States (POMS)	10.69	19.22	4.54	17.99	-1.70	.092

Insomnia Severity (ISI)	7.25	5.63	7.01	4.60	-0.24	.807

Sleepiness (ESS)	7.69	3.85	8.59	3.67	1.23	.220

Sleep Quality (PSQI)	4.14	2.95	5.40	2.56	2.38	**.019**

Chronotype (MEQ)	8.24	3.52	8.44	3.96	0.20	0.84


* p < .05; Bolded p values are statistically different between the ADA genotypes.

To test the secondary hypothesis that ADA genotype would alter psychological health measures, we conducted a series of t-tests examining differences between ADA genotype on the PSS, CESD, STAI- State, STAI- Trait, MEQ, and POMS measures. As seen in ***Table 1***, no significant differences were observed for the PSS, state or trait anxiety, and POMS scores despite the AG allele carriers reporting numerically higher scores than the AG allele carriers. As noted earlier, the genotype groups do not differ on chronotype (MEQ).

To further test the hypothesis that ADA genotype may alter psychological health, we examined the correlations between melatonin and the psychological health variables in each genotype separately. As can be seen in ***Table 2***, melatonin was negatively correlated with PSQI Global scores for the AG allele carriers but not for the GG allele carriers. The difference between these correlations did not reach statistical significance, *z* = 1.85, *p* = .063, 95% CI difference = [–.02, .68]. Melatonin levels were unrelated to the other measures of self-reported sleep.

**Table 2:** Correlations between Melatonin, Sleep, and Psychological Health Variables by Genotype. GG below the diagonal; AG above the diagonal.


	MELATONIN	PSS	STAI STATE	STAI TRAIT	CESD	PSQI	POMS	ESS	ISS

Melatonin		–.077	–.203	–.022	–.222	–.404**	–.296	–.095	–.165

PSS	–.111		–.348*	.712****	.423**	–.259	.125	–.057	**–.221**

STAI State	–.029	.455****		**–.182**	.241	.377*	.325*	–.116	.462**

STAI Trait	–.034	.678****	.605****		.407*	–.310	.043	–.085	**–.231**

CESD	–.097	.694****	.484****	.655****		.408*	.451**	–.039	.499**

PSQI	–.050	.053	.011	.044	.655****		.203	.040	.151

POMS	.052	.315**	.508****	.373***	.362**	–.015		.103	.425**

ESS	.143	.205	.016	.186	.128	.218	.122		.037

ISS	.077	.298**	**.183**	.293*	–.237*	.080	.254*	.499***	


* p < .05, ** p < .01, *** p < .001, **** p < .0001. Bolded correlations are statistically different between the ADA genotypes.

The strength of the relationships between the measures of stress, anxiety, and depression appears to be stronger among the GG allele carriers. As seen in ***Table 2***, significant positive relationships were observed between State Anxiety and Trait Anxiety, State Anxiety and CESD, and PSS and State Anxiety in the GG allele carriers. However, the relationships between State Anxiety and Trait Anxiety, and State anxiety and CESD were nonsignificant in the AG allele carriers. A significant negative relationship between PSS and State anxiety was observed in the AG allele carriers. The difference in the strength of the correlation between PSS and State anxiety was statistically significant, *z* = 4.09, *p* < .0001, 95% CI difference = [ 0.423, 1.106]. State and trait anxiety were significantly related in the GG allele carriers, but not AG allele carriers. The difference in the strength of the correlation between state anxiety and trait anxiety was also statistically significant, *z* = 4.24, *p* < .0001, 95% CI difference = [ 0.414, 1.110]. The differences between the two allele groups on the correlations between Trait anxiety and CESD, *z* = 1.668, *p* = .095, 95% CI difference = [–0.04, 0.584], was in the same pattern with a numerically stronger correlation in the GG allele carriers than the AG allele carriers but the difference did not reach statistical significance. Similarly, the correlations between scores on the POMs and PSS, State Anxiety, and Trait Anxiety were numerically higher in the GG allele carriers than the AG allele carriers, but the correlations were not statistically significantly different from each other.

Differences between the two allele carrier groups also occurred for the strength of the relationships with self-reported sleep measures. A positive correlation was observed in the GG genotype between ESS and ISI scores, while no relationship was observed in the AG group. The difference in the strength of these correlations was statistically significant, *z* = 2.48, *p* = .014, 95% CI difference = [ 0.089, 0.823]. A positive correlation between PSS and ISI scores was observed in the GG group no relationship was observed in the GG group. This difference was statistically significant, *z* = 2.59, *p* = .009, 95% CI difference = [ 0.125, 0.862]. Similarly, a positive correlation was observed in the GG group, but not the AG group, for the relationship between ISI and Trait anxiety scores. This difference was statistically significant, *z* = 2.61, *p* = .009, 95% CI difference = [0.129, 0.867].

## Discussion

Our findings support the central hypothesis that ADA A allele carriers have higher evening melatonin levels. This finding makes empirical sense since ADA A allele carriers express lower enzymatic activity than the homozygous G allele carriers. Lower ADA expression would result in less adenosine degradation, thus heterozygous AG individuals have higher adenosine levels [4,5,6]. Furthermore, increased adenosine levels lead to the synthesis of the melatonin precursor, NAS, from ATP in the pineal gland, which could lead to increased synthesis of melatonin where ASMT is not inhibited [12]. Although a previous study found that salivary melatonin levels did not differ significantly between ADA genotypes, this study was possibly underpowered with 24 participants in each group and results showed a statistical trend (*p* = 0.07; *n* = 12 AG, 12 GG) [39].

The combined effects of high levels of melatonin and adenosine, which both have central roles in the sleep wake cycle (inducing sleepiness as levels rise, then promoting wakefulness as levels drop), could explain our finding of better sleep quality in the AG allele carriers relative to the homozygous GG group. The AG group had an average PSQI score of 4.14 while the GG group had an average PSQI score of 5.4. Given that a PSQI score greater than 5 is indicative of poor sleep quality/significant sleep disturbance, these data suggest that the GG genotype possibly drives carriers towards clinical sleep disturbances. Our finding the AG (higher adenosine) group has higher melatonin levels than the GG group and that melatonin only relates to better sleep quality in the AG group agrees with previous work has shown that melatonin administration improves sleep quality in clinical [40,41,42] and non-clinical [43,44] populations. Notably, the relationship between sleep health and ADA genotype appears limited to Sleep Quality as there were no significant differences observed in our measures of Insomnia (ISI) or overall sleepiness (ESS).

Previous findings of the mood modulating effects of adenosine receptor A2A have been discovered. The deletion or binding of A2A, as demonstrated with caffeine and A2AR blockade (adenosine antagonists), reversed mood and synaptic dysfunction in rats suffering from chronic unpredictable stress [45]. We did not show any effects of ADA genotype on self-reported perceived stress (PSS), anxiety (STAI), or depressive symptomatology (CESD). We do, however, find that the strength of the statistical relationships between the measures of stress, anxiety, and depression is stronger for the GG allele carriers. For example, in the GG group we found robust interactions between State Anxiety and Trait Anxiety, State anxiety and CESD, and PSS and State Anxiety. In the AG allele carriers, however, State Anxiety was not related to either Trait Anxiety or CESD. Relative to the GG homozygotes, high levels of adenosine in ADA A allele carriers may be expected to bind A2A receptors more freely, thus alleviating psychophysiological distress. Unsurprisingly, multiple measures of psychological health and mood were highly correlated with each other independent of genotype.

Although we did not see any group differences in self-reported insomnia (ISS), We found that only the GG group (lower melatonin and poorer sleep quality) had a significant positive correlation between insomnia symptoms and stress and between insomnia and trait anxiety. Both groups showed a significant positive correlation between trait anxiety and insomnia. Overall, the relationship between insomnia and anxiety as well as insomnia and stress are more pronounced in the GG group. In addition, only the GG group showed a positive correlation between insomnia and self-reported sleepiness. Combined, these findings show a unique and robust relationship between sleep and psychological wellness factors in GG carriers.

It is important to note that the measures of sleep health were limited to self-report in this initial study. Given our findings relating ADA genotype with sleep quality, these results warrant further investigation with overnight polysomnography with concomitant melatonin measurements. Moreover, there are myriad lifestyle factors not measured in the current study that relate to our study variables. We cannot be certain that there were not group differences in these measures. For example, exercise is known to improve sleep and sleep depth [46,47]. Body fat percentage also relates to the sleep variables in the current study [48]. In addition, we cannot be certain if there were group differences in typical caffeine use which could also influence our outcome variables [49]. Another consideration in the current study is that previous work showing the relationship between adenosine and melatonin release was conducted in rats. While these findings demonstrated that adenosine signaling increases NAS, there was a differential release of melatonin synthesis via ASMT inhibition. Our data suggest that this differential relationship is not apparent in humans. Finally, this study was composed of mostly female participants (70%) and it is possible our results are mostly applicable to women. However, our data show the same directionality between genotypes on outcome measures in males and females with the exception of perceived stress (see supplemental Table 1).

## Conclusions

To the best of our knowledge, this is the first study to demonstrate a relationship between the ADA SNP rs73598374 with evening melatonin levels and sleep quality. ADA A allele carriers (higher adenosine levels) associated with higher levels of evening melatonin and reported better sleep quality than the homozygous G allele carriers. Moreover, we also find differential relationships between sleep and psychological health between the genotype groups- this which may reveal novel insights about the development of genotype-specific progression of various psychological disorders such as chronic anxiety and stress.

## Data Availability Statement

Data supporting reported results are available upon request: email *tartar@nova.edu*.

## Additional File

The additional file for this article can be found as follows:

10.5334/jcr.209.s1 Supplementary file. Table 1.
