# Supplementary material for: A Functional Adenosine Deaminase Polymorphism Associates with Evening Melatonin Levels and Sleep Quality
Source: J Circadian Rhythms. 2021 Apr 28;19:5. doi: 10.5334/jcr.209 (PMC8086720; doi:10.5334/jcr.209)
Supplement: Supplementary file. — Table 1. [file jcr-19-209-s1.pdf]

Supplemental Table 1. Genotype difference separated by sex.

Variable		Men		Women	
		Mean	SD	Mean	SD
Melatonin	GG	19.8700	10.52342	20.7245	7.85711
	AG	28.7644	28.28441	25.1301	10.37843
PSQ	GG	5.2600	2.79926	5.4644	2.46865
	AG	3.4583	3.38532	4.2647	2.90307
ISI	GG	5.7200	4.94571	7.6346	4.34326
	AG	8.5000	7.47663	7.0294	5.35121
ESS	GG	8.0800	3.60463	8.8365	3.70707
	AG	6.5833	4.05483	7.8897	3.83820
MEQ	GG	8.0800	3.60463	8.9434	3.68163
	AG	7.8333	5.34478	8.0882	3.87977
POMS	GG	5.0000	21.94691	4.3269	15.98495
	AG	22.6667	31.16194	8.5152	15.96896
State Anxiety	GG	27.3200	16.80952	25.9216	13.85185
	AG	22.8333	25.45126	23.7188	17.58341
Trait Anxiety	GG	35.2500	8.80341	35.8868	8.09715
	AG	39.3333	8.18942	37.6875	10.12005
CES-D	GG	14.0800	13.75960	14.0192	11.95332
	AG	21.0000	13.19091	17.1250	16.35641
Perceived Stress	GG	11.6800	7.20948	14.4906	6.55633
	AG	20.3333	13.36663	14.4375	9.49682
